# Anti-Inflammatory Effects of Modified Buyang Huanwu Decoction

**DOI:** 10.1155/2020/6458460

**Published:** 2020-01-13

**Authors:** Hyang-Hwa Ryu, Jun-Chul Kang, Uk Namgung, Song-Yi Kim, Ji-Yeun Park

**Affiliations:** ^1^College of Korean Medicine, Daejeon University, Daejeon 34520, Republic of Korea; ^2^Haram Central Research Institute Co., Ltd., Cheongju 28160, Republic of Korea; ^3^College of Korean Medicine, Gachon University, Seongnam 13120, Republic of Korea

## Abstract

**Methods:**

A cytotoxicity assay for BHD was performed using the MTT assay. Following treatment with BHD, mBHD-1, and mBHD-2 in the presence of lipopolysaccharide (LPS), nitric oxide (NO) secretion was detected in cell supernatants using a NO detection kit. The expression of proinflammatory mediators was detected using RT-PCR and western blotting. To verify the mechanism of mBHD, specific inhibitors of JNK (SP600125) or p38 (SB203580) were used for co-treatment with mBHD, and then the changes in NO and nitric oxide synthase (iNOS) were measured.

**Results:**

Both mBHD-1 and mBHD-2 showed greater anti-inflammatory effects than BHD. Both mBHD-1 and mBHD-2 inhibited NO secretion and decreased the expression of IL-1*β*, IL-6, TNF-*α*, and iNOS. Treatment with a p38 inhibitor and a JNK inhibitor in mBHD-1- and mBHD-2-treated cells resulted in inhibition of NO and iNOS.

**Conclusion:**

We provided the first experimental evidence that mBHD may be a more useful anti-inflammatory than BHD. High concentrations or long-term use of BHD may be harmful to inflammatory status. Therefore, the length of treatment and concentration should be considered depending on the targeted disease.

## 1. Introduction

The inflammatory process is considered a major driving force in the ischemic process, brain repair, neuropathic pain, neuronal injury, and gynecological disease [1]. During the inflammatory response, diverse chemical mediators released by activated immune cells have effects on excitability and synaptic function, and these changes can contribute to the initiation and maintenance of persistent neuropathic pain [2, 3]. Cerebral ischemia is accompanied by a marked inflammatory reaction that is initiated by ischemia-induced expression of cytokines, adhesion molecules, and other inflammatory mediators, including prostanoids and nitric oxide [4, 5]. Thus, the control of the inflammatory process is a logical therapeutic target.

Nitric oxide (NO) has bifunctional roles as a free radical biological messenger in inflammatory responses. It improves the blood supply to injured tissue and is secreted as free radicals that are toxic to bacteria but can cause tissue damage by initiating apoptosis and killing otherwise healthy cells [6]. In rodents, NO can both promote [2, 7] and inhibit inflammatory pain [3, 7]. NO is synthesized by nitric oxide synthase (iNOS), which is mainly upregulated in activated immune cells such as macrophages and glial cells. Therefore, NO and iNOS are major molecules that are generated by macrophages and other phagocytes in inflammation [8].

Traditional Chinese and Korean medicine has been prescribed for health protection and disease control for thousands of years in Eastern Asia. The Buyang Huanwu decoction (BHD) is a traditional medicine mainly used to treat circulatory disorders such as cerebral ischemia, angina, and myocardial infarction, as well as multiple neuritis and thrombophlebitis. According to experimental reports, BHD improves blood circulation, controls pain, regenerates neuronal cells [9–11], and protects against the neuronal damage caused by ischemic and oxidative stress [12, 13].

BHD consists of seven kinds of herbs: Radix Astragali (Astragalus), Angelicae gigantis radix, Paeoniae radix rubra, Cnidii rhizoma, Persicae semen, Carthami flos, and *Lumbricus,* all of which are recorded in the Chinese Pharmacopoeia. These ingredients have effects mainly upon improving blood circulation and protecting thrombogenesis. Recently, various versions of modified BHD (mBHD), a combination of BHD and other effective herbal components, have been used for a synergistic therapeutic effect. Previous studies have reported the effects of mBHD on sciatic nerve injury [11], focal cerebral ischemia [14], and vascular dementia [15].

Considering those reports, we developed two types of mBHD and verified their efficacy according to comparative studies of the anti-inflammatory effect. One was manufactured by eliminating *Lumbricus* (renamed to mBHD-1). *Lumbricus* is a genus of earthworm, and among the seven ingredients of BHD is the only animal material. Thus, we omitted this animal-derived ingredient to avoid the possibility of adverse effects.

Another formulation reduced the concentration of Astragalus (renamed mBHD-2). Traditional BHD contains 120 g of Astragalus. Potentially adverse effects of Astragalus have been reported when used in high doses, despite the enhanced immune system, anti-inflammatory, and antioxidant effects. Astragalus can interact with prescription medications, including anti-hypertensives and immune suppressants and can decrease medication efficacies. It can cause bleeding when used with other anticoagulant, antiplatelet, or antithrombotic agents [16]. Some exporters have been concerned that Astragalus is a legume and thus can cause allergic reactions in people with legume allergies (such as beans and peanuts). For these reasons, we investigated the effects of BHD, mBHD-1, and mBHD-2 on NO secretion and inflammation-associated factors and the signaling pathways involved in the inflammatory responses mediated by lipopolysaccharide- (LPS-) exposed macrophages.

## 2. Materials and Methods

### 2.1. Chemicals and Reagents

The composition of BHD and modified-BHD (named as mBHD-1 or mBHD-2) is described in Table 1. The mBHD-1 formulation omitted *Lumbricus* from the other seven herbs comprising BHD, and mBHD-2 reduced the amount of Astragali Radix used, compared with BHD. The mixture of dried ingredients was boiled in purified saline for 2 h and concentrated under vacuum pressure at 700 mm Hg for 15 h, after which it was freeze-dried. The lyophilized powder of BHD and mBHD were then stored at −20°C until use.

The LPS was purchased from Sigma-Aldrich (St. Louis, MO, USA). Primary antibodies against total extracellular signal-related kinase (ERK), phospho-ERK (p-ERK), total N-terminal kinase (JNK), phospho-JNK (p-JNK), p38 mitogen-activated protein kinase (p38-MAPK), phospho-p38 (p-p38), and glyceraldehyde 3-phosphate dehydrogenase (GAPDH) were purchased from Cell Signaling Technology (Danvers, MA, USA). The antibodies against iNOS and Cox-2 were purchased from Abcam (Cambridge, MA, USA). Horseradish peroxidase- (HRP-) conjugated goat anti-rabbit IgG antibody was obtained from Santa Cruz Biotechnology (Santa Cruz, CA, USA).

### 2.2. Cell Line and Treatments

The murine macrophage cell line RAW264.7 was acquired from the Korean Cell Line Bank (Seoul, Republic of Korea) and was cultured in high-glucose DMEM (Gibco BRL, Gaithersburg, MD, USA) supplemented with 10% fetal bovine serum (FBS, Gibco BRL) and maintained at 37°C in a humidified incubator with 5% CO_2_. BHD, mBHD-1, and mBHD-2 were dissolved in phosphate-buffered saline and filtrated. RAW264.7 cells were pretreated with various concentrations of BHD, mBHD-1, and mBHD-2 or co-pretreated with the inhibitors p38 or JNK for 1 h and then were stimulated with LPS (100 ng/mL) for a period of 30 min to 24 h, depending on the experiment.

### 2.3. Cell Viability Assays

The cytotoxic effect of BHD, mBHD-1, mBHD-2, and several inhibitors on RAW264.7 cells was examined using an EZ-cytox assay kit (Daeil Lab Serve Co, Seoul, Republic of Korea) according to the manufacturer's instructions. Briefly, RAW264.7 cells (1 × 10^5^ cells per well) were seeded in 96-well plates and were treated with various concentrations of materials. After 24 h, 10 *μ*L of EZ-cytox was added into each well and then incubated for another 2 h under standard culture conditions. Cell viability was quantified with a spectrophotometer at 450 nm.

### 2.4. Measurement of NO Production

NO secreted from cells was detected using a NO detection kit (Intron Biotechnology, Seongnam, Republic of Korea). Briefly, RAW264.7 cells (1 × 10^5^ per well) were pretreated with various concentrations of BHD, mBHD-1, and mBHD-2, or co-pretreated with SB203580 (a p38 inhibitor) or SP300125 (a JNK inhibitor) for 1 h and then stimulated with 100 ng/mL of LPS for 24 h. Then, 100 *μ*L of conditioned media was mixed with an equal volume of Griess reagent and incubated at room temperature for 10 min. Absorbance values were measured at 560 nm using a plate reader. The nitrate concentration was calculated based on a nitrite standard curve.

### 2.5. Reverse Transcription-Polymerase Chain Reaction (RT-PCR)

The cells (5 × 10^5^ per well) were pretreated with various concentrations of BHD, mBHD-1, or mBHD-2 for 1 h and then stimulated with 100 ng/mL of LPS for 4 h. Total RNA was isolated using TRIzol reagent (Invitrogen, Carlsbad, CA, USA). Total RNA was reverse-transcribed to synthesize cDNA using the GoScript™ RT system (Promega, Madison, WI, USA). For first-strand synthesis, purified total RNA (1 *μ*g) was incubated with oligo dT (0.5 *μ*g/*μ*L) for 5 min at 70°C, followed by addition of buffer containing 4 *μ*L of 5x reaction buffer, 1 *μ*L of dNTP (10 mM each), 3.5 *μ*L of 25 mM·MgCl_2_, 2 *μ*L of RNase inhibitor (40 U/*μ*L) (Promega), and 1 *μ*L of reverse transcriptase (200 U/*μ*L). The reaction volume was 20 *μ*L, and the mixture was incubated for 1 h at 42°C. Synthesized cDNA (500 ng) was amplified using iSTAR Taq polymerase (Intron Biotechnology, Seongnam, Republic of Korea). The primer information is described in Table 2. The amplified DNA was detected on an agarose gel containing Ecodye™ (BIOFACT, Daejeon, Republic of Korea). The data correspond to three independent experiments.

### 2.6. Western Blot Analysis

RAW264.7 cells (1 × 10^5^ per well) were pretreated with various concentrations of BHD, mBHD-1, or mBHD-2 or co-pretreated with SB203580 (a p38 inhibitor) or SP300125 (a JNK inhibitor) for 1 h and then stimulated with 100 ng/mL of LPS for 30 min or 24 h, respectively. For preparation of total protein, cells were lysed in RIPA buffer (50 mM Tris (pH 8.0), 5 mM EDTA, 150 mM sodium chloride, 0.5% deoxycholic acid, 0.1% SDS, 1% NP-40, 1 mM PMSF, and 1 mg/mL protease inhibitor cocktail). The protein concentrations were determined using a Bio-Rad Protein Assay Kit (Bio-Rad, Hercules, CA, USA). Total protein (10 *μ*g) was separated by 8–10% sodium dodecyl sulfate-polyacrylamide gel electrophoresis (SDS-PAGE) and transferred to PVDF membranes (Pall Corp., Hauppauge, NY, USA). The membranes were incubated for 1 h at room temperature with 5% nonfat dry milk, probed overnight at 4°C with each of the primary antibodies, and incubated with horseradish peroxidase-labeled goat anti-rabbit antibody. The signals were detected by enhanced chemiluminescence (ECL, Amersham Biosciences, Buckinghamshire, UK). The data correspond to three independent experiments.

### 2.7. Statistical Analysis

Data were expressed as mean ± standard error of the mean (SEM). The statistical differences between the groups were analyzed by one-way ANOVA followed by Tukey's multiple comparisons test using GraphPad Prism 7.0 (GraphPad Software Inc., CA, USA). A *p* value <0.05 was considered statistically significant.

## 3. Results

### 3.1. Cytotoxicity of BHD, mBHD-1, and mBHD-2

**Figure 1:**
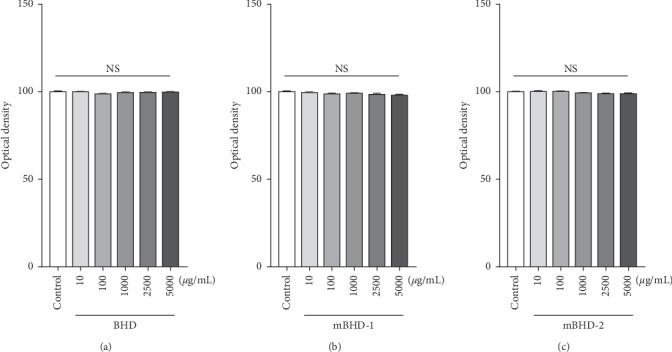
Cytotoxic effects of BHD, mBHD-1, and mBHD-2 on murine macrophage RAW264.7 cells. Cells were treated with 10, 100, 1,000, 2,500, or 5,000 *μ*g/mL of BHD (a), mBHD-1 (b), or mBHD-2 (c), and their cytotoxicity was detected using the MTT assay. Statistical differences were analyzed using one-way ANOVA followed by Tukey's multiple comparisons test. Error bars indicate SEM. NS, not significant.

To determine the suitable concentration of BHD for these studies, we first tested the cytotoxic effect of BHD, mBHD-1, and mBDH-2 on RAW264.7 cells. The cells were treated with various concentrations (10, 100, 1000, 2500, and 5000 *μ*g/mL) of BHD for 24 h. We found that BHD, mBHD-1, or mBHD-2 showed no cytotoxic effects from 10 to 5,000 *μ*g/mL in RAW264.7 cells (Figure 1).

### 3.2. Effect of BHD, mBHD-1, and mBHD-2 on the Production of NO in LPS-Stimulated RAW264.7 Cells

**Figure 2:**
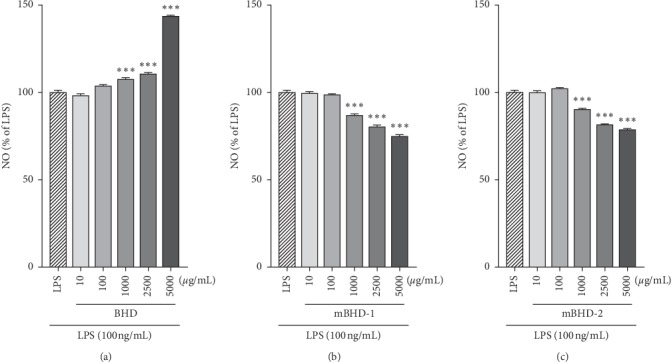
Effects of BHD, mBHD-1, and mBHD-2 on NO production in LPS-exposed RAW264.7 cells. Cells were pretreated with different concentrations of BHD (a), mBHD-1 (b), and mBHD-2 (c) for 1 h and then were stimulated with 100 ng/mL LPS. After 24 h, secreted NO was measured in conditioned media using a NO detection kit. ^*∗∗∗*^*p* < 0.001 vs. LPS. Statistical differences were analyzed using one-way ANOVA followed by Tukey's multiple comparisons test. Error bars indicate SEM.

It is well known that a large amount of NO is produced by macrophages in an inflammatory environment. We investigated whether BHD, mBHD-1, or mBHD-2 could control the NO released by LPS. Both mBHD-1 and mBHD-2 gradually suppressed the NO induced by LPS at 1,000, 2,500, and 5,000 *μ*g/mL (*p* < 0.001). Conversely, BHD-pretreated cells showed the opposite results. Treatment of cells with BHD gradually increased NO at 100, 1,000, 2,500, and 5,000 *μ*g/mL, reaching an approximately 40% increase at 5,000 *μ*g/mL, compared with LPS (*p* < 0.001) (Figure 2).

### 3.3. Effect of BHD, mBHD-1, or mBHD-2 on Morphological Changes in LPS-Exposed RAW264.7 Cells

**Figure 3:**
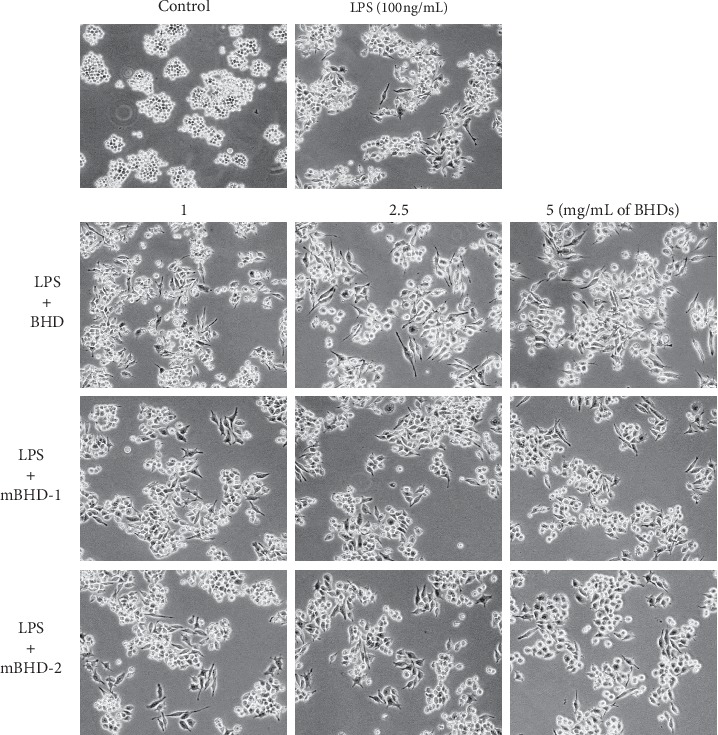
Effects of BHD, mBHD-1, and mBHD-2 on the morphology of LPS-exposed RAW264.7 cells. Cells were pretreated with 1, 2.5, or 5 mg/mL of each treatment, and their morphologic changes were observed using light microscopy.

The morphology of RAW264.7 cells reportedly changes following stimulation with LPS. Thus, we examined whether BHD affected morphologic changes in LPS-stimulated cells. The morphology of LPS-exposed RAW264.7 cells was spindle-like, and the cells pretreated with BHD, mBHD-1, or mBHD-2 were observed to possess similar shapes to LPS-stimulated cells (Figure 3).

### 3.4. Effect of BHD, mBHD-1, or mBHD-2 on the Expression of Proinflammatory Cytokines in LPS-Exposed RAW264.7 Cells

**Figure 4:**
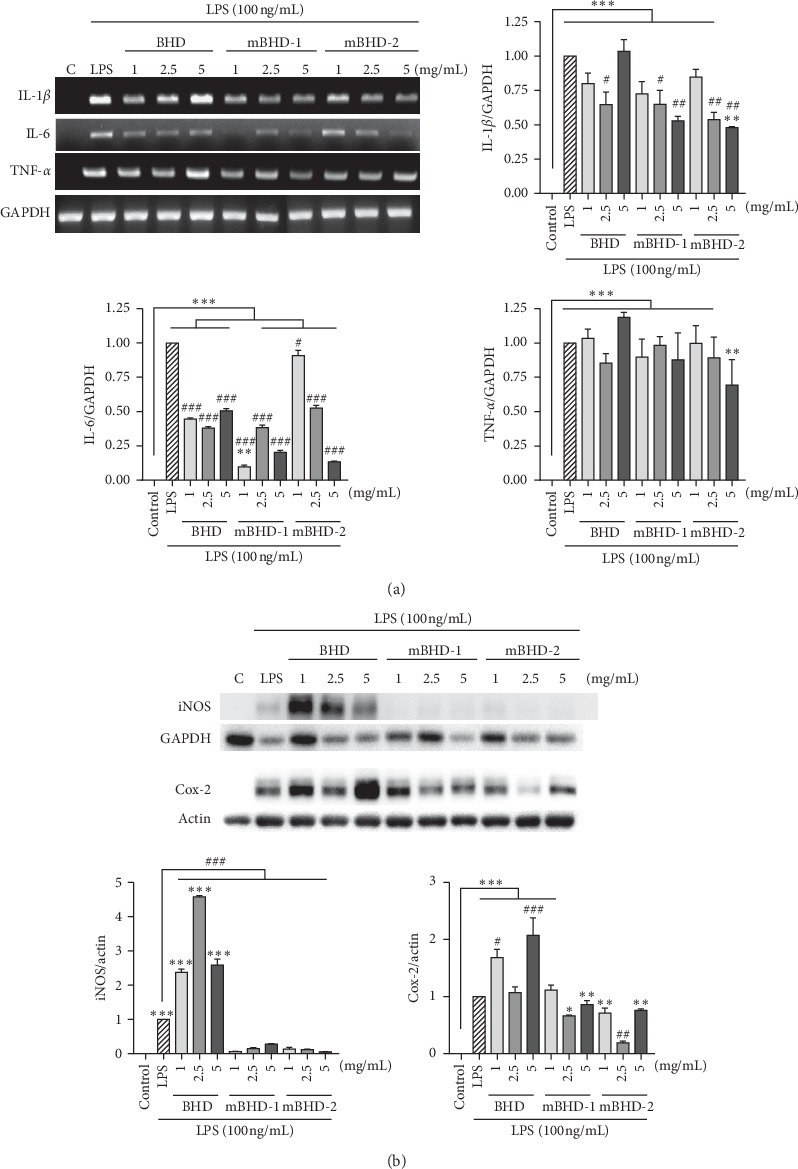
Effect of BHD, mBHD-1, and mBHD-2 on the expression of proinflammatory cytokines in LPS-exposed RAW264.7 cells. Cells were pretreated with 1, 2.5, or 5 mg/mL of each drug for 1 h and then were stimulated with LPS for 4 h. Transcriptional levels of inflammatory cytokines of IL-1β, IL-6, and TNF-α were detected using RT-PCR (a) and the protein expression of iNOS and Cox-2 was detected using western blotting (b). IL: interleukin; TNF: tumor necrosis factor; iNOS: inducible nitric oxide; Cox-2: cyclooxygenase-2; GAPDH: glyceraldehyde 3-phosphate dehydrogenase; LPS: lipopolysaccharide. C, control; ^*∗*^*p* < 005; ^*∗∗*^*p* < 0.01; ^*∗∗∗*^*p* < 0.001 vs. control; ^#^*p* < 005; ^##^*p* < 0.01; ^###^*p* < 0.001 vs. LPS. Statistical differences were analyzed using one-way ANOVA followed by Tukey's multiple comparisons test. Error bars indicate SEM.

To determine whether BHD could regulate proinflammatory mediators, we examined the transcriptional level of proinflammatory cytokines in LPS-exposed RAW264.7 cells. As shown in Figure 4(a), cells pretreated with BHD showed decreased expression of interleukin- (IL-) 1*β* at 2.5 mg/mL (*p* < 0.05), and IL-6 at 1, 2.5, and 5 mg/mL (*p* < 0.001) compared with that of cells stimulated only with LPS. The cells pretreated with mBHD-1 showed decreased expression of IL-1*β* at 2.5 (*p* < 0.05) and 5 mg/mL (*p* < 0.01) and IL-6 at 1, 2.5, and 5 mg/mL (*p* < 0.001) compared to the LPS-treated cells. On the other hand, the cells pretreated with mBHD-2 showed decreased expression of IL-1*β* at 2.5 and 5 mg/mL (*p* < 0.01) and IL-6 at 1 (*p* < 0.05), 2.5, and 5 mg/mL (*p* < 0.001) compared to the LPS-treated cells. mBHD-2 tended to decrease expression of tissue necrosis factor- (TNF-) *α* at 5 mg/mL in a dose-dependent manner, but there was no statistical significance.

Cyclooxygenase- (Cox-) 2 and inducible iNOS are induced by proinflammatory cytokines in an inflammatory environment [17]. We examined the expression of Cox-2 and iNOS using western blotting. Compared to the LPS-treated cells, the expression of iNOS was significantly decreased in the cells pretreated with mBHD-1 and mBHD-2 at every dose (*p* < 0.001), while it was highly induced in the BHD-pretreated cells (*p* < 0.001). The expression of Cox-2 was increased in the BHD-pretreated cells at 1 (*p* < 0.05) and 5 mg/mL (*p* < 0.001) and decreased in the mBHD-2-pretreated cells at 2.5 mg/mL (*p* < 0.01) compared to LPS-treated cells (Figure 4(b)).

### 3.5. Effect of mBHD-1 and mBHD-2 on Activation of p38, JNK, and ERK1/2 Pathways in LPS-Exposed RAW264.7 Cells

**Figure 5:**
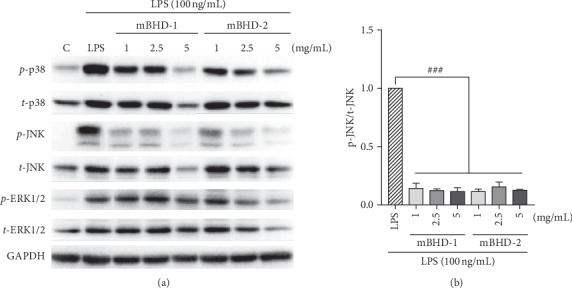
Effects of BHD, mBHD-1, and mBHD-2 on the activation of the JNK, p38, and ERK1/2 pathways in LPS-exposed RAW264.7 cells. Cells were pretreated with 1, 2.5, or 5 mg/mL of each drug for 1 h and then were stimulated with LPS for 30 min. The expression of target factors was detected in total protein using western blotting (a). Protein expression of p-JNK was most markedly inhibited by mBHD-1 and mBHD-2 (b). LPS: lipopolysaccharide; GAPDH: glyceraldehyde 3-phosphate dehydrogenase; ERK: extracellular signal-mediated kinase; JNK: c-Jun N-terminal kinase; p38: p38 mitogen-activated protein kinase. C, control; ^###^*p* < 0.001 vs. LPS. Statistical differences were analyzed using one-way ANOVA followed by Tukey's multiple comparisons test. Error bars indicate SEM.

p38, JNK, and ERK play an important role in inflammatory responses such as iNOS expression. To elucidate the molecular mechanism of the anti-inflammatory effects of mBHD-1 and mBHD-2, we examined the activities of ERK, p38, and JNK using western blotting. The activation of p38 and JNK was decreased in mBHD-1 or mBHD-2 pretreated cells, compared with LPS-stimulated cells. However, mBHD-1 and mBHD-2 did not affect the activation of the ERK pathway in LPS-stimulated cells (Figure 5).

**Figure 6:**
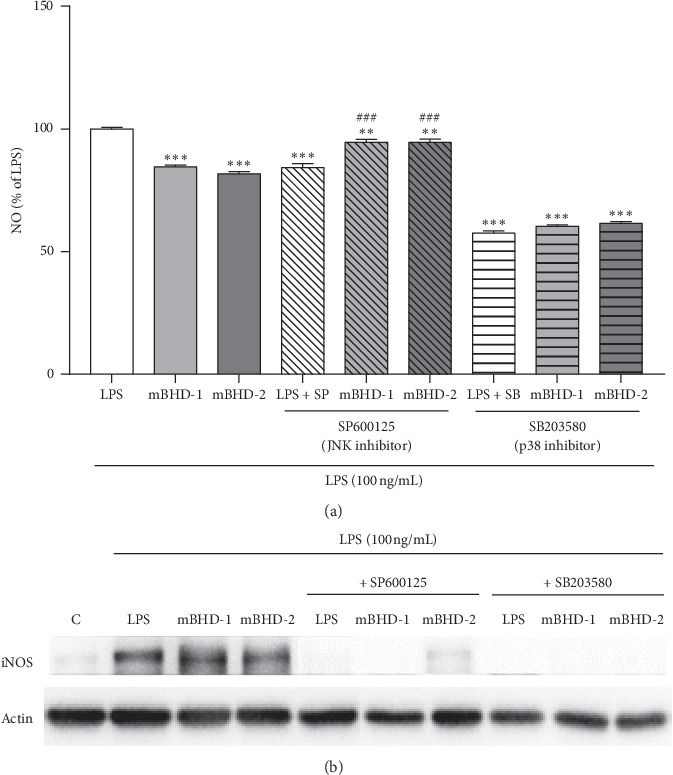
Both mBHD-1 and mBHD-2 inhibit NO secretion by regulating the p38 signaling pathway in LPS-exposed RAW264.7 cells. Cells were pretreated with SP600125 (a JNK inhibitor) or SB203580 (a p38 inhibitor) with or without BHD, mBHD-1, or mBHD-2 for 1 h and then were stimulated with LPS (100 ng/mL). After 24 h, NO was detected in conditioned media using a NO detection kit (a), and expression of iNOS and Cox-2 was measured in total protein using western blotting (b). LPS + SP: LPS and SP600125 treatment; LPS + SB: LPS and SB203580 treatment. ^*∗∗*^*p* < 0.01; ^*∗∗∗*^*p* < 0.001 vs. LPS; ^###^*p* < 0.001 vs. LPS + SP. Statistical differences were analyzed using one-way ANOVA followed by Tukey's multiple comparisons test. Error bars indicate SEM.

**Figure 7:**
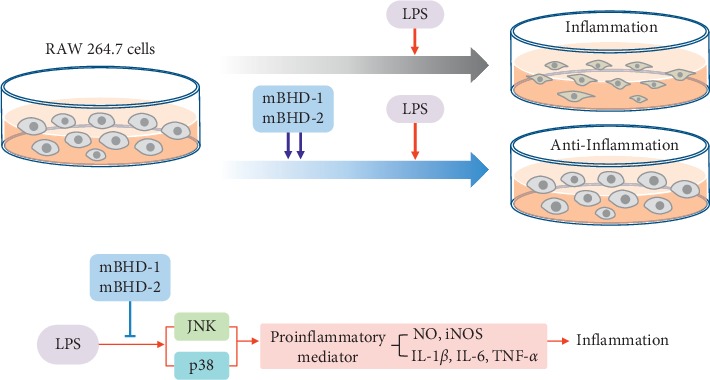
Summary of anti-inflammatory effects of mBHD-1 and 2.

To confirm whether mBHD-1 or mBHD-2 regulate NO and iNOS through stimulate the JNK and p38 signaling, we co-treated cells with a JNK inhibitor (SP600125) or a p38 inhibitor (SB203580) with mBHDs. After treatment of LPS-stimulated cells with SP600125 alone, NO was reduced by approximately 20%. When cells were co-treated with LPS and mBHD-1 (or mBHD-2), NO increased by approximately 15% compared with that in cells treated with SP600125 alone (*p* < 0.001). In cells treated with SB203580 alone, NO was reduced by about 40%, and the NO reduction between the SB203580 treatment alone and the SB203580 + mBHD-1 (or mBHD-2) treatment showed similar results, compared with that in cells treated with LPS alone (Figure 6(a)). The expression of iNOS was significantly decreased in both SP600125- and SB203580-treated cells, compared with that of the control group (Figure 6(b)). These results indicated that mBHD-1 and mBHD-2 regulate NO and iNOS expression through the p38 MAPK and JNK pathway (Figure 7).

## 4. Discussion

Traditional BHD consists of seven natural herbal components that function to improve the circulatory system and immunity in humans [18–20] and protect neural activity and regeneration [21, 22]. Thus, this traditional medicine has been prescribed mainly to patients with autoimmune encephalomyelitis [23] or ischemic brain disorders [12, 24] for more than 300 years. Recent studies on the use of various mBHD, which have reduced BHD components or are combined with other effective herbs, have provided reports regarding their therapeutic advantage. In a rat model of Alzheimer's disease, mBHD with a reduced quantity of all components improved memory deficiency and decreased the cerebral infarction in the hippocampus [25, 26]. Another version of mBHD with added Polygalae radix and Acori Graminei Rhizoma showed anti-inflammatory and antioxidant effects on microglial cells [20]. Two studies using modified BHD with Achyranthis Radix, Acori Graminei Rhizoma, Polygalae Radix, Cinnamomi Ramulus, and Salviae Miltiorrhizae Radix reported reduction of the cerebral infarction size and neurological deficit in ischemia/reperfusion models [14] and hippocampal inflammatory responses in vascular dementia models [15].

A BHD preparation including Lycopi Herba, Typae pollen, Trogopterori feces, and Corydalis tubers showed antithrombotic activities and can be applied to diverse female diseases caused by thrombosis and inflammation such as endometriosis, myoma, and chronic pelvic inflammatory disease [27]. According to a clinical report in 2011, BHD without *Lumbricus* improved symptoms in patients with hyperhidrosis resulting from sequela of spinal cord injuries. Tritici Fructus Levis is utilized mainly for inhibiting spontaneous perspiration and night sweats by securing the exterior.

Astragalus not only has various therapeutic effects, including enhanced immune system function as well as anti-inflammatory and antioxidant effects, but also poses potential risk when used at a high dose [28–31]. *Lumbricus* is an earthworm genus considered to possess therapeutic effects in epilepsy and stroke [32]. Until now, the side effects of *Lumbricus* have not been reported, but out of the seven ingredients composing BHD, this represents the only animal-derived component, whereas the other components of BHD are plant-derived.

Based on these reports, we developed two types of mBHD, the mBHD-1 preparation (without *Lumbricus*) and the mBHD-2 preparation (reduced amount of Astragalus) and verified the improvement of anti-inflammatory effects. We compared the anti-inflammatory effects of BHD and mBHDs in LPS-stimulated murine macrophage RAW264.7 cells. The activity of the iNOS and NO system is mediated by macrophages and other phagocytes during inflammatory episodes [8]. In immune-defense status, iNOS is activated by proinflammatory cytokines such as IL-1*β*, TNF-*α*, IFN-*γ*, and LPS, resulting in large amounts of NO release.

On the basis of those reports, we examined the effect of BHD, mBHD-1, and mBHD-2 on changes in NO secretion and expression of iNOS and proinflammatory cytokines in LPS-stimulated RAW264.7 cells. According to our results, the cells treated with mBHD-1 and mBHD-2 showed significantly and dose-dependently decreased NO secretion and expression of IL1-*β*, TNF-*α*, Cox-2, and iNOS. The results of mBHD-1 and mBHD-2 treatments showed similar results. However, BHD-treated cells showed opposite results. BHD increased NO secretion and the expression of these factors. As mentioned above, BHD is composed of ingredients that are useful in blood circulation.

NO has bifunctional roles as a free radical biological messenger and a modulator of inflammatory responses; these roles can be separated as defensive mechanisms and tissue damage mechanisms. Furthermore, NO improves the blood supply to injured tissue and is secreted as a free radical that is toxic to bacteria and modulates the actions of macrophage-derived cytokines on target cells. However, NO can cause tissue damage by inducing apoptosis [33]. In an oxidative environment, high levels of NO released by iNOS have the opportunity to react with superoxide, leading to peroxynitrite formation and cell toxicity. These properties may define the roles of iNOS in host immunity, enabling its participation in antimicrobial and antitumor activities as part of the oxidative burst produced by macrophages [34]. Proinflammatory cytokines (TNF-*α*, IL-6, and IL-1*β*) are important mediators in the inflammatory process and are mainly produced by activated macrophages [35]. Excessive production of these proinflammatory cytokines will lead to clinical problems associated with autoimmune disorders (rheumatoid arthritis) and inflammatory response syndrome (septic shock) [36].

To further investigate the mechanism of the anti-inflammatory effects of mBHD, we examined major signaling molecules associated with the inflammatory process. LPS-stimulated macrophages have been widely reported to activate three different groups of MAP kinases: ERK, JNK, and p38 [37]. Our results showed that BHD, mBHD-1, and mBHD-2 significantly reduced p38 and JNK activated by LPS but did not affect ERK1/2 phosphorylation. Activation of p38 and JNK inhibited by mBHD-1 and mBHD-2 was stably maintained 24 h later but that inhibited by BHD was recovered in 24 h.

Finally, we confirmed whether mBHD-1 and mBHD-2 control NO secretion and iNOS expression through the regulation of the p38 and JNK pathway using the inhibitors SB203580 for p38 and SP600125 for JNK in LPS-induced RAW264.7 cells. SB203580 is a selective inhibitor of p38 [38], and SP600125 is a potent, selective, and reversible inhibitor of JNK [39]. As our results demonstrate, the cells co-treated with mBHD and SB203580 showed significantly reduced NO and iNOS expression in the presence of LPS, compared with those exposed to LPS alone. The inhibitory effects on NO secretion produced by SB203580 were higher than those produced by SP600125.

## 5. Conclusion

In conclusion, mBHD-1 and mBHD-2 were more effective in suppressing the NO/iNOS system following activation by LPS through the regulation of the p38 and JNK pathway in LPS-exposed RAW264.7 cells. Both mBHD-1 and mBHD-2 showed greater anti-inflammatory activity than BHD. These results may indicate that the prescribed concentration of Astragalus in BHD should be determined depending on the target disease. This study provided the first experimental evidence of an anti-inflammatory effect of mBHD. However, further studies are still needed to elucidate the fundamental mechanism of mBHD in various types of *in vitro and in vivo* disease models. Moreover, further research should continue to develop various combinations of BHD and develop specific prescriptions for each targeted disease.

## Figures and Tables

**Table 1:** Raw materials of BHD, mBHD-1, and mBHD-2.

Raw materials	BHD	mBHD-1	mBHD-2
Astragali Radix	120 g	120 g	20 g
Angelicae gigantis radix	6 g	6 g	6 g
Paeoniae radix	4.5 g	4.5 g	4.5 g
Lumbricus	3 g	—	3 g
Cnidii rhizoma	3 g	3 g	3 g
Persicae semen	3 g	3 g	3 g
Carthami flos	3 g	3 g	3 g

BHD, Buyang Huanwu decoction; mBHD, modified Buyang Huanwu decoction.

**Table 2:** Primers used in this study.

Target	Primer sequence
GAPDH	F. caggatgaggacatgagcac
	R. ctctgcagactcaaactcca
IL-1*β*	F. caggatgaggacatgagcac
	R. ctctgcagactcaaactcca
IL-6	F: ggaaattggggtaggaagga
	R: ccggagaggagacttcacag
TNF-*α*	F. cagccttgtcccttgaagagaacc
	R. tactgaacttcggggtgattggtc

GAPDH, glyceraldehyde 3-phosphate dehydrogenase; IL, interleukin; TNF, tumor necrosis factor.

## Data Availability

The data used to support the findings of this study are available from the corresponding author upon request.

## References

[1] Iadecola C., Anrather J. (2011). The immunology of stroke: from mechanisms to translation. *Nature Medicine*.

[2] Ellis A., Bennett D. L. H. (2013). Neuroinflammation and the generation of neuropathic pain. *British Journal of Anaesthesia*.

[3] Schomberg D., Ahmed M., Miranpuri G., Olson J., Resnick D. K. (2012). Neuropathic pain: role of inflammation, immune response, and ion channel activity in central injury mechanisms. *Annals of Neurosciences*.

[4] Iadecola C., Alexander M. (2001). Cerebral ischemia and inflammation. *Current Opinion in Neurology*.

[5] Liu Y., Lin R., Shi X. (2011). The roles of buyang huanwu decoction in anti-inflammation, antioxidation and regulation of lipid metabolism in rats with myocardial ischemia. *Evidence-Based Complementary and Alternative Medicine*.

[6] Pavanelli W. R., Nogueira Silva J. J. (2010). The role of nitric oxide in immune response against trypanosoma cruzi infection∼!2009-10-14∼!2010-01-25∼!2010-04-07∼!. *The Open Nitric Oxide Journal*.

[7] Duarte I. D., dos Santos I. R., Lorenzetti B. B., Ferreira S. H. (1992). Analgesia by direct antagonism of nociceptor sensitization involves the arginine-nitric oxide-cGMP pathway. *European Journal of Pharmacology*.

[8] Choi W. S., Jeong J. W., Kim S. O. (2014). Anti-inflammatory potential of peat moss extracts in lipopolysaccharide-stimulated RAW 264.7 macrophages. *International Journal of Molecular Medicine*.

[9] Ha J. C., Kim Y. H., Woo W. H., Nam S.-Y. (2001). Promotion of nonspecific cytotoxic T lymphocyte activity by Bo-yang-hwan-oh-tang. *Korean Journal of Pharmacognosy*.

[10] Chen A., Wang H., Zhang J. (2008). BYHWD rescues axotomized neurons and promotes functional recovery after spinal cord injury in rats. *Journal of Ethnopharmacology*.

[11] Cheng Y. S., Cheng W. C., Yao C. H. (2001). Effects of buyang huanwu decoction on peripheral nerve regeneration using silicone rubber chambers. *American Journal of Chinese Medicine*.

[12] Mu Q., Liu P., Hu X., Gao H., Zheng X., Huang H. (2014). Neuroprotective effects of Buyang Huanwu decoction on cerebral ischemia-induced neuronal damage. *Neural Regeneration Research*.

[13] Yang S., Gao Q., Xing S. (2011). Neuroprotective effects of Buyang Huanwu decoction against hydrogen peroxide induced oxidative injury in Schwann cells. *Journal of Ethnopharmacology*.

[14] Choi Y., Kim S.-K., Choi I.-Y. (2011). Amelioration of cerebral infarction and improvement of neurological deficit by a Korean herbal medicine, modified Bo-Yang-Hwan-O-Tang. *Journal of Pharmacy and Pharmacology*.

[15] Oh T. W., Jung H. W., Park Y. K. (2015). Effect of modified Bo-yang-Hwan-o-Tang, a polyherbal medicine on the hippocampal neuronal damage in a rat model of global ischemia. *Pharmacognosy Magazine*.

[16] Lu W. I., Lu D. P. (2014). Impact of Chinese herbal medicine on american society and health care system: perspective and concern. *Evidence-Based Complementary and Alternative Medicine*.

[17] Seibert K., Masferrer J. L. (1994). Role of inducible cyclooxygenase (COX-2) in inflammation. *Receptor*.

[18] Son H. Y., Park Y. K. (2010). Neuroprotective effect of modified Boyanghwano-Tang and the major medicinal plants, Astragali Radix and Salviae Miltiorrhizae Radix on ischemic stroke in rats. *Korea Journal of Herbology*.

[19] Chen J., Ding Y., Yan X. (2011). Comparative pharmacokinetics of three marker compounds in mBHT and single-herb extract after oral administration to rats. *Journal of Pharmaceutical and Biomedical Analysis*.

[20] Kim H. J., Lee S. R. (2008). The effects of Kakam Boyang Hwanoh-tang (KBHT) and PalMihapChongMung-tang (PMCMT) on protecting microglia and inhibiting acetylcholinesterase and oxidants. *Journal of Oriental Neuropsychiatry*.

[21] Kim K. J., Namgung U. (2018). Facilitating effects of Buyang Huanwu decoction on axonal regeneration after peripheral nerve transection. *Journal of Ethnopharmacology*.

[22] Chang I. A., Lim H. D., Kim K. J., Shin H., Namgung U. (2016). Enhanced axonal regeneration of the injured sciatic nerve by administration of Buyang Huanwu decoction. *Journal of Ethnopharmacology*.

[23] Ma C. G., Wang X. Q., Li Y. H. (2017). Buyang Huanwu decoction exhibits therapeutic potential in experimental autoimmune encephalomyelitis (EAE) by the inhibition of ROCK/TLR-4/NF-*κ*B inflammatory signaling pathway. *Journal of the Neurological Sciences*.

[24] Hao C. Z., Wu F., Shen J. (2012). Clinical efficacy and safety of buyang huanwu decoction for acute ischemic stroke: a systematic review and meta-analysis of 19 randomized controlled trials. *Evidence-Based Complementary and Alternative Medicine*.

[25] Seo S. H., Jung I., Lee S. R. (2008). The effects of Kakam Boyang Hwanoh Tang (KBHT) hot water extract & ultra-fine powder on the alzheimer’s disease model. *Journal of Oriental Neuropsychiatry*.

[26] Han C., Park Y. (2010). The effect of modified boyanghwano-tang on the brain infarction through the anti-apoptosis of neuronal cells in ischemic rats. *Journal of Acupuncture Research*.

[27] Jung Eun L., Dong Youl Y. (2006). Experimental study on anti-thrombotic and anti-inflammatory effect of kami-Boyang Hwanoh-tang. *Journal of Physiology & Pathology in Korean*.

[28] Auyeung K. K., Han Q.-B., Ko J. K. (2016). Astragalus membranaceus: a review of its protection against inflammation and gastrointestinal cancers. *The American Journal of Chinese Medicine*.

[29] Chu D. T., Wong W. L., Mavligit G. M. (1988). Immunotherapy with Chinese medicinal herbs. II. Reversal of cyclophosphamide-induced immune suppression by administration of fractionated Astragalus membranaceus in vivo. *Journal of Clinical and Laboratory Immunology*.

[30] Yang G., Cheng W. D., Zhang L. F., Xing W. T., Zhou Y. Y., Sun X. F. (2014). Comparative study on substituting hedyseri radix for astragali radix in serum containing yiqiyangxue prescription on aged mice spleen lymphocyte proliferation and anti-oxidant effect. *Journal of Chinese Medicinal Materials*.

[31] Ryu M., Kim E. H., Chun M. (2008). Astragali Radix elicits anti-inflammation via activation of MKP-1, concomitant with attenuation of p38 and Erk. *Journal of Ethnopharmacology*.

[32] Liu C. H., Lin Y. W., Tang N. Y., Liu H. J., Huang C. Y., Hsieh C. L. (2012). Effect of oral administration of Pheretima aspergillum (earthworm) in rats with cerebral infarction induced by middle-cerebral artery occlusion. *African Journal of Traditional, Complementary and Alternative Medicines*.

[33] Hibbs J. B., Taintor R. R., Vavrin Z., Rachlin E. M. (1988). Nitric oxide: a cytotoxic activated macrophage effector molecule. *Biochemical and Biophysical Research Communications*.

[34] Mungrue I. N., Husain M., Stewart D. J. (2002). The role of NOS in heart failure: lessons from murine genetic models. *Heart Failure Reviews*.

[35] Zhang J. M., An J. (2007). Cytokines, inflammation, and pain. *International Anesthesiology Clinics*.

[36] Chen L., Deng H., Cui H. (2017). Inflammatory responses and inflammation-associated diseases in organs. *Oncotarget*.

[37] Downey J. S., Han J. (1998). Cellular activation mechanisms in septic shock. *Frontiers in Bioscience*.

[38] Cuenda A., Rouse J., Doza Y. N. (1995). SB 203580 is a specific inhibitor of a MAP kinase homologue which is stimulated by cellular stresses and interleukin-1. *FEBS Letters*.

[39] Bennett B. L., Sasaki D. T., Murray B. W. (2001). SP600125, an anthrapyrazolone inhibitor of Jun N-terminal kinase. *Proceedings of the National Academy of Sciences*.

